# Answering the ultimate question “What is the Proximal Cause of Aging?”

**DOI:** 10.18632/aging.100525

**Published:** 2012-12-30

**Authors:** Mikhail V. Blagosklonny

**Affiliations:** Department of Cell Stress Biology, Roswell Park Cancer Institute, BLSC, L3-312, Elm and Carlton Streets, Buffalo, NY 14263, USA

**Keywords:** MTOR, TOR, rapamycin, senescence, cancer, stroke, development, growth

## Abstract

Recent discoveries suggest that aging is neither driven by accumulation of molecular damage of any cause, nor by random damage of any kind. Some predictions of a new theory, quasi-programmed hyperfunction, have already been confirmed and a clinically-available drug slows aging and delays diseases in animals. The relationship between diseases and aging becomes easily apparent. Yet, the essence of aging turns out to be so startling that the theory cannot be instantly accepted and any possible arguments are raised for its disposal. I discuss that these arguments actually support a new theory. Are any questions remaining? And might accumulation of molecular damage still play a peculiar role in aging?

It is commonly believed that aging is caused by random accumulation of molecular damage due to failure of maintenance, because repair is costly [1, 2]. As emphasized by Kirkwood, “the aging process is caused by the gradual buildup of a huge number of individually tiny faults - some damage to a DNA strand here, a deranged protein molecule there, and so on” [2]. The view is very logical, intuitive and simple. As argued recently [3], the scholastic philosopher William of Ockham would surely have liked it. Yet, the damage/repair theory leads to incorrect predictions and to bizarre paradoxes [4, 5]. Also, the free radical version of this theory has not been confirmed [6-15]. After all, William of Ockham lived before Galileo. Now we know that a theory must make correct predictions and be useful, rather than just be elegant.

What if aging is not caused by accumulation of molecular damage? What if random accumulation of molecular damage is irrelevant to aging? Then the cause of molecular damage is not really important. What if aging does not start from day one. In fact, the mortality rate is lower in 10-years old children than in infants. So the period of growth is hardly aging. But when developmental growth is finished, growth-signaling pathways may continue to run on inertia (Fig. 1). Where would that lead the soma?

**Figure 1:**
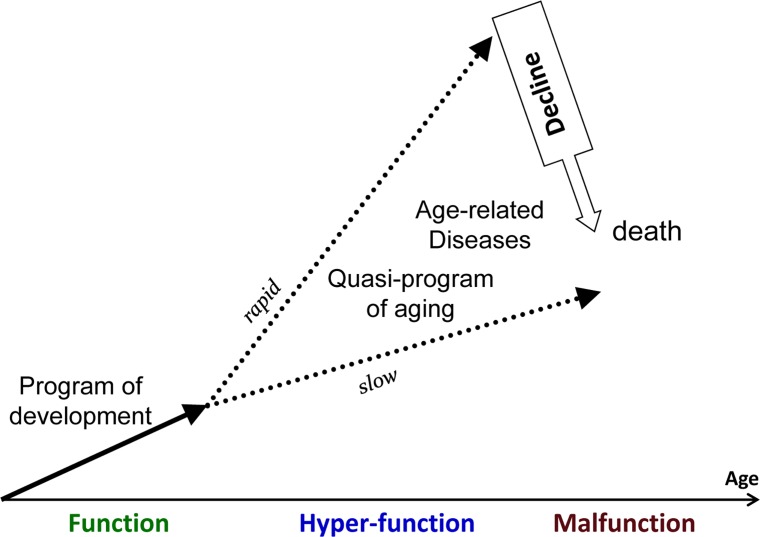
Aging as a quasi-program of development When development is finished cellular normal functions become excessive (hyperfunctions). They lead to diseases. Development is strictly programmed and therefore is precise (one line), whereas aging is not (a continuation of developmental program or quasi-program), and so age-related diseases (ADR) occur at different age.

## The hyperfunction theory

In cell culture, when actual proliferation is blocked, then still active growth-signaling and nutrient-sensing pathways such as the TOR (Target of Rapamycin) pathway cause senescence [16-27]. TOR can convert quiescent cells into senescent cells without any involvement of molecular damage [28-36]. The TOR pathway is involved in yeast and organismal aging from worm to mammals [37-53] as well as in age-related disease in mammals [54-65]. The same pathway, which drives developmental growth, later drives aging and its associated diseases. As discussed in detail previously [54, 66], aging is of course not a program, but it is a quasi-program, a useless and unintentional continuation (or run on) of developmental programs. Similarly, cellular senescence is a continuation of cellular growth [36, 67, 68]. In brief, over-stimulation leads to increased functions. Such functions include secretion by fibroblasts, contraction by arterial smooth muscle cells (SMC), aggregation by platelets, bone resorption by osteoclasts, lipogeneisis by fat cells, glycogenesis by liver cells, inflammation by neutrophils, phagocytosis by macrophages and so on and so one. Also, overstimulation may render cell signal resistance due to feedback block of signaling pathways. In turn, hyperfunction coupled with signal-resistance causes loss of homeostasis, diseases, organ damage and eventually death of the organism [54]. For example, taken together, hyperfunctions of arterial smooth muscle cells, macrophages, hepatocytes, fat cells, blood platelets, neurons and glial cells, fibroblasts, beta-cells cause organ hypertrophy and fibrosis, atherosclerosis and hypertension (and their complications such as stroke and infarction), osteoporosis and (as complication, born rupture), age-related blindness, gangrenes, renal and heart failure and even cancer. There is no ARD that cannot be linked to initial cellular hyperfunction, in part, driven by mTOR [54]. The senescence-associated secretory phenotype (SASP) [69- 73] is a characteristic hyperfunction of fibroblasts (and some other cells) caused by hyper-mitogenic stimulation of arrested cells [74, 35]. Chronic inflammation, a classic example of hyperfunction, is associated with aging and age-related diseases [75- 82]. Even telomere shortening [83-93] can also be viewed as a consequence of hyperfunction insofar as it is promoted by hyper-proliferation, and perhaps, therefore, is associated with accelerated age-related diseases (ARD). Although loss of functions is often in terminal aging, loss of function always results from initial hyperfunction (and no another example could be found [54]. This is also applicable to simple multicellular organisms such as Drosophila and C. Elegans [94, 95].

Given that cellular hyperfunction is one of the main characteristics of aging, David Gems, Yila de la Guardia and Linda Partridge suggested a short name “hyperfunction theory” [94, 95], which I will use here.

Many predictions of the hyperfunction theory have already been confirmed [96]. Pro-aging signal-transduction pathways and potential anti-aging agents that target them have been revealed, including several existing drugs such as rapamycin and metformin [97]. Moreover, inhibition of hyperfunction in downstream processes regulated by aging pathways, e.g. attenuation of protein synthesis, extends lifespan [98-100]. Intriguingly, some anti-hypertensive drugs “calm down” hyper-functional signaling-pathways, simultaneously preventing other age-related diseases such as cancer (see for references [101]). Examples include inhibitors of beta-adrenergic [101-103] as well as of angiotensin II signaling [104, 105], which are both linked to mTOR signaling [106]. Metformin, an anti-diabetic drug, which indirectly inhibits the mTOR pathway, decreases cancer incidence, prevents premature menopause and increases lifespan in rodents [107-112]. Rapamycin not only delays typical age-related diseases in animal models but also extends life span in mice [113-119]. Thus, there exists the opportunity to extend both health span and lifespan in our life time [120, 121].

## The molecular damage theory dies hard

But what about the molecular damage? It was assumed that molecular damage contributes to aging because it accumulates with time. Well, over time you may accumulate money in your bank account. However, neither accumulation of molecular damage nor accumulation of money is a cause of your aging. Yes, molecular damage must accumulate. But although molecular damage accumulates, it does not necessarily limit lifespan, particularly if other causes limit life span. By analogy, if everyone died from accidents, starvation and infection early in life, then aging and age-related diseases (such as obesity and atherosclerosis) would not even be known. By the same token, “aging” due to molecular damage will not manifest itself, if aging due to hyperfunction invariably limits life span [122]. Notably, as a marker of hyperfunction in senescent cells, DNA damage response-signaling pathways can be hyper-activated even without DNA damage [123-126].

**Figure 2:**
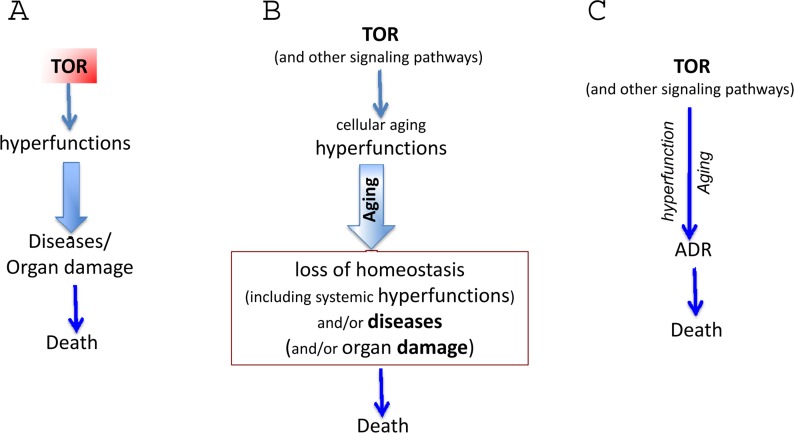
The hyperfunction theory: three representations (**A**) The simplest model. Cause-effect relationship between TOR-driven hyperfunction and death via age-related diseases (diseases). For diseases, we mean age-related diseases (ADR). (**B**) Extended model. Diseases include initial loss of homeostasis and systemic hyperfunction (an increase in blood pressure and glucose) leading to organ damage like stroke, menopause and diabetes. Cellular hyperfunctions (e.g. hyper-secretion) can be viewed as cellular aging. (**C**) Unification of the hyper-function theory. Since cellular aging is cellular hyperfunction, it can be unified with systemic hyperfinction. In brief, aging = hyperfunction. Loss of homeostasis, decline and organ damage, which can be unified as ADR (age-related DISEASES).

The hyperfunction theory suggests that repair of molecular damage is important for long life, exactly because it is harmful from day one. But the importance of any process for viability does not imply its role in aging. For example, although DNA replication is important, it (or its abnormalities) does not drive aging. Nonetheless, with a few exceptions, most gerontologists cannot let go of the damage accumulation theory, historically the dominant paradigm in the field. This outlook is superbly expressed by Piotr Zimniak, who argues that the molecular damage theory cannot be replaced by the hyperfunction theory [3] He has briefly summarized the hyperfunction theory in figures 1 A (here figure 1A). First, I will extend figure 2 from A to B (Fig. 2 A,B) in part because the terms “loss of homeostasis” and “age-related diseases” are manifestations of aging either according to molecular-damage or to hyperfunction theories, retrospectively. “Loss of homeostasis” and “age-related diseases” are not alternatives, but instead overlapping terms, almost synonyms, closely related phenomena.

## Loss of homeostasis and age-related disease and death

Cellular hyper-function (and secondary signal resistance) must cause loss of homeostasis and, eventually, organ damage and death. Loss of homeostasis encompasses pre-diseases (e.g., glucose intolerance or insulin-resistance), syndromes such as metabolic and diseases such as hypertension, obesity, diabetes, atherosclerosis, renal and cardiac failure and so on. Obesity, hypertension, hyperlipidemia, hyperglycemia, hyperinsulinemia, hyperprolactinemia (and other *hypers*) are all systemic hyperfunctions, measurable by standard medical methods. They are systemic manifestations of cellular hyperfunction. Such systemic hyperfunctions also constitute loss of homeostasis.

Yet, given my medical background, I prefer the term “disease” to “loss of homeostasis”. Otherwise patients with blindness due to diabetes and patients with stroke due to hypertension and atherosclerosis would both be just suffering from loss of homeostasis. Second, dividing “loss of homeostasis” into specific diseases allows us to better apply the wealth of biomedical knowledge. It is known in detail how hyperfunctions such as an increased lipogenesis by fat and liver cells, hyper-aggregation of platelets, increased contractility and hypertrophy of arterial smooth muscle cells, migration and phagocytosis by macrophages - after many steps that are very well-known in pathology and medicine – cause, for example, damage to the brain via stroke. The sequence of events from TOR-driven hyperfunction to atherosclerosis, type II diabetes and menopause were recently discussed [58, 59, 63, 127-130] or will be discussed soon.

**Figure 3:**
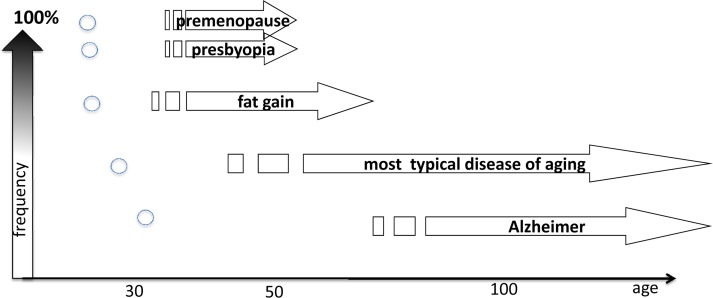
The relationship between the onset of age-related disease (ADR) and it frequency The onset of the ADR determines its frequency and the precision of the onset. Arrows: Age of age-related diseases and its frequency in general population. Small circles: Genertic variants of the similar disease, which is a rare case in general population. High frequency is only (!) in very small groups of genetically-abnormal patients.

Age-related diseases (ARD), or if you prefer “loss of homeostasis (LOH)” limit lifespan in protected environments. ARD can be delayed by slowing down aging due to deactivation of the mTOR pathway by calorie restriction, genetic manipulation and drugs. The incidence of age-related diseases increases exponentially with aging. ARD include cancer, hypertension, atherosclerosis, diabetes type II, osteoporosis, sarcopenia, Alzheimer and Parkinson diseases, macular degeneration, organ fibrosis and hypertrophy and many more. Their complications (and often an immediate cause of death) include stroke, infarction, ventricular fibrillation, complications of diabetes such as renal failure, and so on. Also, menopause (in females) and presbyopia are normal age-related diseases, whose incidence does not increase indefinitely since almost all are affected by age 55 (Fig. 3). And of course “cosmetic diseases” such as baldness can be linked to hyper-function too, but this is a topic for another article.

I combine the terms “loss of homeostasis and/or disease” (and call them ADR in this article; may be another term could be malignant hyperfunction or even malfunction). Mild and brief loss of homeostasis is not necessarily called disease but they are not lethal anyway. Some forms of damages such as hip fracture due to osteoporosis are not viewed as loss of homeostasis. Therefore, “and/or” (Fig. 2B). Organ damage or organ malfunctions could occur either early or late in the pathogenesis of ADR. There are positive-feedback loops between cellular hyperfunction and loss of homeostasis as well as vicious cycles between “loss of homeostasis” and “organ damage”. Organ damage is the end point: cardiac arrest and stem brain damage always happen as an immediate cause of death. But all that is well described in the medical literature. All these late and terminal events MUST be the same regardless of either the “molecular damage” or the “hyperfunction theories. In contrast, gerontology is concerned whether it is “molecular damage” or “hyperfunction” cause malfunctions and diseases (causes of death). And the molecular damage theory cannot explain hyperfunctions.

For brevity, I will use the term disease, which include loss of homeostasis (biomedical term) and non-random organ damage (Fig. 2C). Also, TOR causes cellular and system hyperfunctions (e.g., hyperlipidemia and hypertension) are continuation of cellular hyper-functions. Cellular hyperfunction is the essence of cellular aging. The consequence of hyperfunction is the organismal aging, defined as a progressive increase in death rate. Therefore, in figure 1 C, cellular and systemic hyperfunction are combined, as an essence and equivalent of aging, which drives loss of homeostasis, diseases, macro-damage and secondary decline (all combined as “disease” or malfunction in figure 3C).

Finally, TOR-driven hyperfunctions are not only involved in diseases but also in “cosmetic” age-related alterations/diseases. Male pattern of baldness is often associated with hyper-stimulation with testosterone. I will not discuss cosmetic alterations in detail here. Many of them are deadly in some environmental conditions. For example, loss of teeth or vision is deadly in the wild. These are conditional diseases, so I will include them in the term “disease” or ARD.

For complex organisms like mammals the relationships between hyperfunctions (aging), diseases and damage (decline) are:

1. Hyperfunctions (increased cellular functions) including hypertrophy are primary. This is the essence of aging, which silently causes malfunctions and age-related diseases (ADR).
2. Decline of functions, malfunctions and atrophy are secondary. For example, hyper-stimulation of beta-cells by nutrients and mitogens can cause its apoptosis. Here is important to emphasize however that apoptosis can be also a form of hyperfunction, unneeded continuation primary function such as apoptosis during development of the immune system.
3. Damage is caused by aging, not the reverse.
4. Damage is not molecular. It is macro-damage (tissue, system and organ damage), like stroke, infarction, metastases, broken hip fracture and renal failure. Damage may take a form of sudden “catastrophe”, even though hyperfunctional aging slowly generates diseases for decades. If a patient survives infarction (due to medical intervention), she can live for many years, reflecting the fact that catastrophe was not due to the burden of molecular damage. In small organisms (e.g., Drosophila) organ can consist of one cell or even cell's part, but still an organ.

Diseases and organ damage are not random. There is a limited number of common causes of death. This is because hyperfunction is a continuation of develop-mental function, not a random process. Stroke, infarction and cancer are common. Cessation of thrombocytopoiesis (for example) is not. In C elegance and humans, “age-related diseases” are very different of course, because physiology and anatomy is so different [131]. For example, nutrient-sensitive organ (“hypothalamus”) contains 2 neurons only. These two neurons mediate diet-restriction-induced longevity in C. elegans [132]. In all multi-cellular organisms, the rule is: function, then hyperfunction (aging), then mal-function.

**Argument 1:** As argued by Piotr Zimniak, “I would hesitate to accept that catastrophic events, such as a stroke in a middle-aged person or sepsis in an otherwise healthy individual, are aging” [3].

Me too. To say that “aging causes diseases” is not to mean that “all diseases are caused by aging”. Clearly, not all diseases are caused by aging. For example, sepsis is caused by microorganisms. Even “age-related” diseases and their complications such as stroke could result from genetic defects, developmental abnormalities, environmental factors and so on. Or stroke could be due to accelerated atherosclerosis and hypertension, which in turn could be due to either accelerated aging, genetic/environmental factors or both. It could be a combination of genetic defects, environmental factors (smoking) and aging, which drives atherosclerosis, platelet hyperfunction and hypertension. For details here, I refer the readers to medical textbooks.

**Figure 4:**
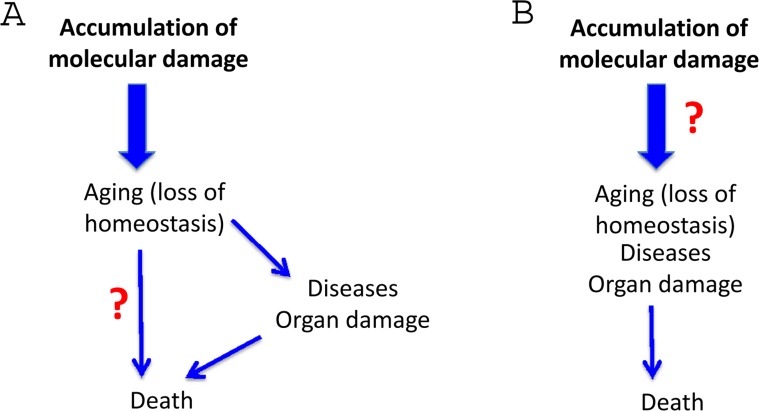
The molecular damage theory Elimination of the repetitive terms. (**A**) Repetitive links between aging and death. (**B**) The only link between aging and death.

The point is not that age-related diseases are always caused by aging but that aging is *sufficient* to cause age-related diseases (either organ hyperfunction or its malfunction/failure). Even without genetic predisposition and environmental causes, aging causes stroke and other diseases, which together kill every human being (so far) by the age of 122 (the age at death of the oldest woman, Jeanne Calment). True age-related diseases (ARD) are manifestations and exacerbations of the normal aging process. Any particular ARD (age-related diseases) may not happen in any given individual simply because other ARD can terminate life first (Fig. 4). As we will discuss, time of onset, the frequency and the inevitability of ARD vary because aging is not programmed but an imprecise continuation of development. But here is a second argument against the hyperfunction theory.

**Argument 2:** Aging kills not by causing catastrophic damage but “-rather loss of homeostasis, i.e., aging, can lower cell/tissue robustness and precipitate catastrophic events” [3].

In other words, by lowering robustness “accumulation of molecular damage” in the brain would precipitate stroke. This scenario is imaginary. This is not how it happens. Rather hyperfunctions such as hypertension, propensity to thrombosis, rapture of or stenosis by atherosclerotic plaque cause stroke. By analogy, hurricanes cause damage due to their “hyperfunction and robustness”. In contrast, weakness of construction does not precipitate catastrophe in good weather. Let us extend the analogy. An increased use of oil and coal (purposeful program) leads to carbon emissions, which lead to global warming (aimless quasi-program, analogous to hypertension, atherosclerosis and hyper-coagulation), which contribute to other factors that generate a particularly powerful storm, which can be damaging, if it strikes a vital location such as New Orleans (or the brain, in the case of stroke).

Should we re-invent medical science to fit the theory of molecular damage? More plausibly, it is any theory of aging that should reconcile itself with physiology, pathology and medicine, rather than vice versa. Hyperfunction (hypertension, propensity to thrombosis, etc) causes catastrophic events. Even cancer cells, which actually accumulate molecular damage, are robust and in turn damage and kill the organism due to their robustness. Only after non-random system/organ damage, is there decline and weakness. The decline phase is not driven by TOR and is only marginally quasi-programmed, a process run loose. This is a subject for emergency medicine, not gerontology.

Elderly patients who are immobilized by stroke, for example, are vulnerable to infections and sepsis. However, it is not aging per se that provoked sepsis but is rather immobolization caused by stroke (or in other words complications of age-related diseases, which then take their own TOR-independent course). But even then hyperfunctions contribute to deadly outcome such as fatal septic shock, which can be prevented with rapamycin [133].

## Normal and hyper-functions

Hyper-functions result from the continuation (or running on) of normal functions. For example, blood pressure rises from birth to adulthood. This developmental program increases robustness by assuring optimal blood pressure. But its continuation (hyperfunction) leads to hypertension. As another example, at puberty in girls, a carefully-regulated increase of estrogen and gonadotropin levels switch on the reproduction (program, function). A continuation of the same process (quasi-program, hyperfunction) progressively impairs fertility after 30 (Fig. 2) and eventually culminates in ovarian failure and menopause [128, 134, 135]. Then, levels of estrogens drop (decline), accelerating osteoporosis. Menopause is a typical age-related disease [136]. It is not called a disease simply because it happens in all women (Fig. 3). Actually, it does not: some women die before menopause. Just 300 years ago most women died before menopause. Menopause is a quasi-programmed disease [128]. Menopause is particularly program-like, because it happens relatively early in life, when quasi-program (hyperfunction) is still very directional, a precise continuation of the developmental program for reproduction.

I need to emphasize that hyper-function is not always an increased function. It may be unneeded normal function like growth and apoptosis. In analogy, a car that is driving at 65 pmh at small parking lot is “hyperfunctional”, even if at the highway, this speed isr normal. Similarly, the TOR activity that is constantly and chronically at the level of rapidly dividing cells, (“proliferating cell” level) is gerogeni in resting postmitotic cells.

## Some facts on age-related diseases (ARD)

Any theory of aging (regardless of the cause of aging) must be in agreement with the following medical facts:

1. The incidence of age-related diseases (ARD) increases exponentially with age in parallel with death rate. This is not co-incidental but rather reflects the fact that ARD cause death. If an individual does not die from one disease (e.g., cancer), he/she will eventually die from another (e.g., stroke and myocardial infarction). Of course, small organisms, which even lack the heart have different ARD, which would be better studied, if these small animals “complain to their “doctors”.
2. Aging kills via diseases. In fact, no one has ever died without a cause. And “natural causes” always denotes disease. In very elderly people, the diagnosis of “death from natural causes” means many competing causes (diseases) simultaneously. Death from natural causes simply excludes death from suicide and homicide. But there is no such medical diagnosis as “death from accumulation of molecular damage”. This “nonexistent diagnosis” is simply not needed because one or several deadly diseases can be always (always) found (unless homicide). Even sudden cardiac death usually results from myocardial fibrillation (archetypical hyper-function of electrical myocytes andventricular hypertrophy).

The later the onset of a disease, the greater the variability in the time of its onset (Fig. 2). This is because aging is not programmed, but quasi-programmed. It is a continuation of development. Development is strictly regulated. But aging and its diseases are not because they are an unintended, non-guided continuation of developmental growth. By analogy, the longer you walk with your eyes closed, the less precisely you continue in the same direction. The further from the end of development, the bigger deviation and the higher imprecision. A particular age-related disease (ARD) could strike at 40 and at 110 and at 300 in some people, if they lived so long). It would strike everyone, if other diseases would not terminate their lives first (Fig. 2). It is perhaps the case that any age-related disease would develop sooner or later in anyone, and only death from other diseases precludes death from any given one.

The time of onset of ARD determines the frequency and inevitability of the disease (Fig. 2). Importantly, this also correlates with the invariability of age of disease in everyone and its precision. Early ARD is a direct continuation of developmental programs. I already mentioned progressive loss of fertility and menopause in women, which is a direct continuation of reproductive function. Another example is presbyopia, a progressive hardening of lens that prevents focusing at close small objects [137, 138]. Symptoms such as problems focusing on fine print, requiring glasses, are noticed between the ages of 40 and 50, very often almost suddenly. This ARD is a quasi-program, a continuation of developmental program. The near point of vision is very close in infants and then progressively moves further away as an organism grows larger. The same process continues later in life: from 7 cm at age 10, to16 cm at age 40, to 100 cm at age 60. Mechanistically, this is hyperfunction due to progressive increase of thickness and stiffness of the crystalline lens as well as continual growth of the lens [139, 140]. Most importantly, a continuation of this quasi-program (quasi-quasi-program) is age-related nuclear cataract, which is a cause of blindness much later in life, involving genetic and environmental factors [141]. Therefore, cataract does not happen to everyone and is variable in its timing of onset.

**Argument 3:** “it would be difficult to identify catastrophic death events in, for example, bacteria, organisms that also age”.

Yes, I agree of course. If bacteria indeed age, they may age from accumulation of molecular damage, precisely because they do not undergo hyperfunctional aging and have a chance to experience (or not) aging from accumulation of damage. But multicellular organisms do not die from the same “aging” as bacteria. In multicellular organisms, hyperfunctions lead to eventual disintegration of the soma. In contrast bacteria may age from accumulation of moleculer damage (most probable) but they have no multicellular soma and never dir from stroke anyway. Bacteria is irrelevant example of commom aging.

## Age-related diseases in worm and flies

Due to extensive biomedical research, humans are the most studied animal. No one dies from “healthy aging”, without a cause: either natural causes such as disease or homicide/suicide. Similar, all mammals die from age-related diseases (ARD), albeit their frequencies vary dramatically. This could be expected given a quasi-programmed nature of aging and ARD. If one disease occurs earlier than other diseases, it will mask all other diseases. Likewise, Pacific Salmon die from quasi-programmed ADR, namely massive organ damage, resulting from continuation of the reproductive program. This is a particular clear example of quasi-programmed hyperfunction [142]. It is often stated that all Pacific Salmon die from aging/ADR, and therefore that this is a program. Not true. Only 1-2% Pacific Salmon die from ADR, all others die earlier from accidental causes, which must be expected in the wild [142], (see figure 3 in ref.[4]).

Fruit flies and worms die from manifestations of aging or ARD. ADR as causes of death are more specific than vague death from aging. In fruit flies, diseases include neurodegeneration [143-146], cardiac dysfunction [147, 148] and even “diabetes” [149]. TOR is involved in age-related pathologies in flies [150, 46, 47, 150]. Insulin, which activates TOR, is implicated in pathologies resembling mammalian metabolic syndrome [53, 151], diet-induced obesity, diabetes and cardiac dysfunction [152-156]. As in mammals, high-fat-diet-induced obesity and heart dysfunction are regulated by the TOR pathway [147]. Thus main pro-aging pathways in *Drosophila* include insulin/FOXO/TOR, TOR, JNK, NF-kB [46, 47, 145, 147, 157-162].

In the nematode worm *Caenorhabditis elegans*, several “diseases” can be identified [163-173], including infections [174]. Many of the diseases of aging seen in worms are consistent with quasi-programmed hyperfunction, and within its short 2-3 week lifespan C. elegans develops a number of pathologies involving extreme hypertrophy [94, 95]. One example, discussed by David Gems and Linda Partridge [94], involves yolk, which is synthesized in the intestine in large quantities to provision the developing oocytes. After several days of reproduction, reproduction ceases. However, production of yolk continues, and consequently it accumulates in large pools within the body cavity. This accumulation is suppressed in long-lived daf-2 insulin/IGF-1 receptor mutants. Moreover, inhibition of yolk protein gene expression extends worm lifespan, suggesting that quasi-programmed yolk accumulation increases age-related mortality [94]. So worm and fly die from ADR too, just different ADR. Needless to say that even different mammals have different frequency of common ADR. Needless to say that even different nations have different prevalence of ADR.

**Argument 4:** If aging is quasi-programmed hyper-function, why then is aging associated with decline and atrophy? The critic agrees that “atrophy, a classical sign of aging-related decline, can be in fact secondary to an initial hyperfunction and hypertrophy (Blagosklonny, 2012).” This is correct: “signs of age-related decline” are secondary.

## From hyperfunction/hypertrophy to decline/atrophy

There are diverse mechanisms of secondary atrophy during aging.

1. Quasi-programmed (hyperfunction-driven) apoptosis. In this case, atrophy is secondary to signal resistance due to mTOR overactivation. For example, in insulin-secreting beta-cells overactivated by nutrients and insulin, mTOR causes cellular hypertrophy/hyperplasia/hyperfunction and secondary insensitivity to IGF-1 and deactivation of Akt, leading to beta-cell death [130, 175, 176]. mTOR-dependent hyperfunction and hypertrophy of beta-cells, may eventually culminate in cell loss and decline of function [177, 178]. Similar quasi-programmed apoptosis could be observed in the muscle, the immune system and subcutaneous adipocytes. Apoptosis is programmed in development but quasi-programmed in aging. Also, strong hyper-mitogenic drive can force post-mitotic neurons into the cell cycle leading to apoptosis in Alzheimer disease [179-184]. Importantly, cellular senescence is associated with both hyper-mitogenic drive and death in mitosis, explaining this phenomenon [124].
2. Hyper-stimulation-driven cell exhaustion. For example, mTOR overactivation [68, 185, 186] or growth factor stimulation [187] drives exhaustion of stem cells and ovarian oocytes [188-191]
3. Poor wound-healing could be due to signal resistance secondary to cellular aging and hyperglycemia [192].
4. Metabolic-self destruction due to hyper-active TOR. [108, 193, 194]
5. Common atrophy is secondary not to aging itself but to age-related diseases. This is disease-driven atrophy, the end point of some diseases. This is so far away from initial cause that it is completely unrelated to aging and is mTOR-independent. Let me provide two examples. Atherosclerosis of the femoral artery can cause not only atrophy but even gangrene of the feet. Atrophy is very common in ischemia due to atherosclerosis. As another example, hip fracture in an elderly person often leads to prolonged immobilization. Muscle atrophy is secondary to immobilization, which is secondary to the broken hip, which is secondary to osteoporosis, which is secondary to hyperfunction of osteoclasts… and so on. This is disease-driven atrophy. Atrophy is common because it is secondary to diseases. This further supports the thesis that there is no healthy aging (healthy aging is no aging or very slow aging).

**Argument 5:** When cellular hyperfunction causes atrophy, there must be molecular mechanisms such as interaction of ligand with receptor, protein aggregation and so on. More specifically, the critic claims that “an overproduced ligand may over stimulate or desensitize a receptor” [3] For example, an overproduced ligand may over stimulate or desensitize a receptor, and an overabundant protein may aggregate and interfere with intracellular trafficking, or co-precipitate with and thus withdraw essential cell constituents. First, this is still an example of hyper-function. Importantly, rapamycin alleviates toxicity of different aggregate-prone proteins [195] and decreases aggregate-prone proteins [196-200]Second, this is not molecular damage but signal transduction. In contrast, the molecular damage theory is about life long accumulation (!) of random (!) molecular damage due to failure of repair/maintenance (!). As emphasized by Kirkwood, “the aging process is caused by the gradual buildup of a huge number of individually tiny faults - some damage to a DNA strand here, a deranged protein molecule there, and so on” [2].

If we redefine “signal transduction” as “accumulation of random molecular damage due to failure of maintenance”, then yes, this is a cause of aging and age-related diseases. Then driven by mTOR, “molecular damage” (formerly, signal transduction) includes protein phosphorylation as well as protein synthesis, inhibition of autophagy and caspase activation. But this is hyperfunction, not failure of *maintenance*. Phosphorylation of S6K by mTOR, modification of NF-kB or dephosphorylation of Akt, for instance, are not molecular damage. This is signal transduction. Exactly the same signal transduction is involved in development, cell growth, differentiation and apoptosis. These are normal functions. Since the same molecular events are involved in development and developmental growth, this would lead to the *reductio ad absurdum* that development is caused by damage. More plausibly, their (developmental functions) continuation gives rise to quasi-programmed hyperfunction. These normal functions and hyperfunctions can be inhibited by signal transduction inhibitors including rapamycin.

Life long accumulation of molecular damage is irrelevant to aging. Aging (hyper-function of signaling pathway) causes damage and this damage is organ/system/organismal damage (not molecular damage). Hyperfunction of liver cells, for instance, after several decades, contributes to brain damage via stroke.

## Healthy death in molecular damage theory

As commonly depicted [3], aging (loss of homeostasis), caused by molecular damage, in turn causes death via two independent ways (Fig. 4A). The first way is via an increased susceptibility to diseases. The second way is directly without any diseases, and is the true aging mechanism, according the molecular damage theory. This is incorrect. Consider young healthy constructor worker fall to death from the storm (Yes, this is “healthy” death but not from aging). Another example. The fall from the height of 100 year old person is due to either age-related Parkinson's disease or due to infarction. This is not death from healthy aging. This is death due to an age-related disease.

**Figure 5:**
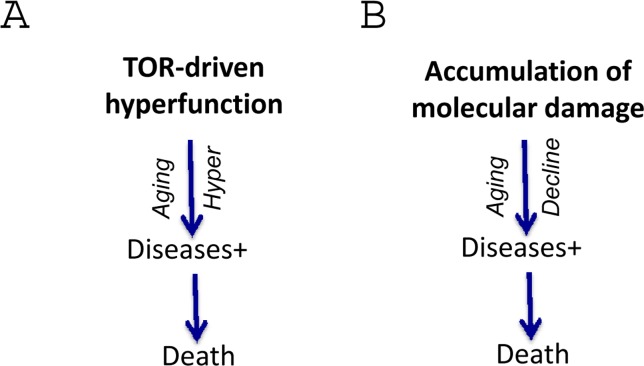
Harmonizing two theories for direct comparison The causes of molecular damage are mostly unknown and also irrelevant.

**Figure 6:**
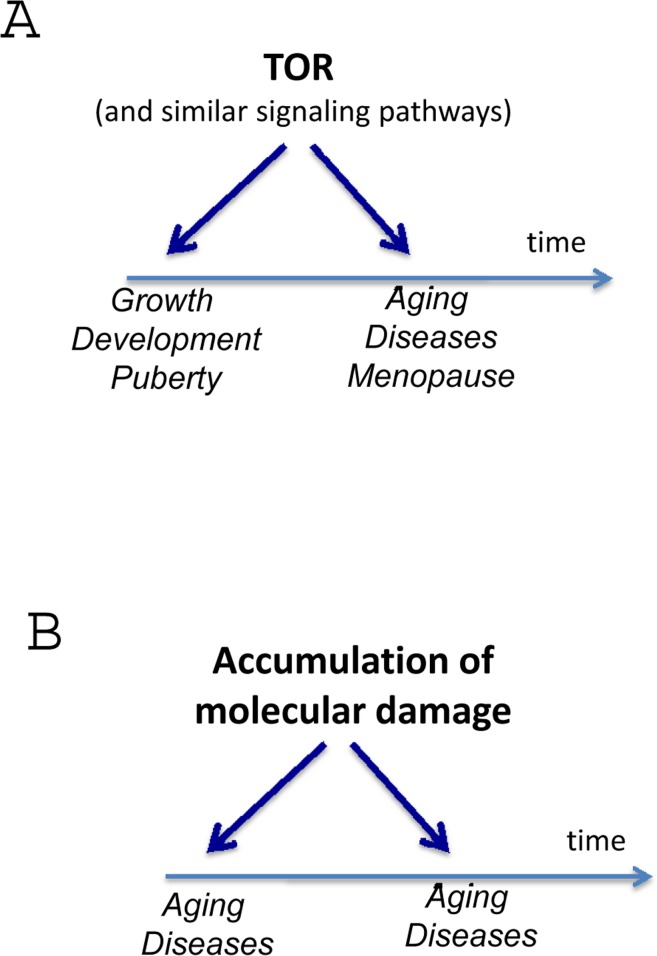
Harmonizing two theories for direct comparison The differences are obvious.

As we discussed, death from aging is death from diseases (natural causes) (Figs. 5,6). Even the oldest people do not die from healthy aging. There is no such medical diagnosis as healthy death or death from asymptomatic accumulation of molecular damage. Of course, we can consider “loss of homeostasis” broadly, including severe deviations of homeostasis or diseases. But then there is no other “disease pathway” anyway. Regardless of the causes of aging, the causes of death and the path from “loss of homeostasis” to death are well known. In all theories of aging, this must be identical because this is a medical fact (Fig. 5). There is no death directly from healthy aging (of healthy loss of homeostasis). This is a part of the same path (Fig. 5). So, shift from A to B (Fig. 4), exactly as in figure 2 B.

Now the question is how accumulation of molecular damage drives each age-related diseases (Figs. 5, 6). For example, how accumulation of molecular damage causes insulin resistance, hypertension and obesity. There is no obvious answer.

## Is TOR-driven hyperfunction true aging?

Is it true aging? Or is it just a process related to disease and mortality [3]? No, it is genuine aging. Not only because it determines mortality (a hallmark of aging is an exponential increase in mortality rate) but also because mTOR-dependent hyperfunctions, signal-resistance, hypertrophy, hypermitogenic drive coupled with loss of regenerative potential are markers of cellular senescence. Cellular senescence can be caused by mTOR activation in cell culture. Although by arresting cell cycle, DNA damage response (DDR) creates conditions for senescence (if mTOR is active), the accumulation of molecular damage itself does not cause senescence in cell culture [35, 36]. In contrast, accumulation of molecular damage contributes to cancer cell immortality [122]. Thus TOR-driven hyperfunction links cellular aging to age-related diseases and organismal aging, defined as an increase of the probability of death.

## Do any questions remain unanswered?

Although we have answered most of the issues relating to the damage/maintenance vs hyperfunction debate raised by P. Zimniak (one is left for the conclusion), it may seem that many more unanswered questions remain. Yet, some of them have been answered previously (see PubMed “Blagosklonny” and related references within) and others will be answered in forthcoming articles. The evolutionary aspects, the links between development and aging, pro-aging and anti-aging genes, common signaling pathways that drive aging, cellular senescence and diseases such as cancer have been extensively discussed. According to the hyperfunction theory, aging and its manifestations are never programmed: even menopause and the death of Pacific Salmon are not programmed. Both are excellent examples of quasi-program, a non-adoptive, aimless, harmful continuation of a reproductive program.

Also, as already discussed, lifespan is determined not only by the aging process but also by aging-tolerance, an ability to tolerate disease of aging and their complications. As a matter of fact, almost all medical interventions (including by-pass surgery and teeth proteases) increase aging tolerance rather than slow down aging. When needed, natural selection may favor anatomical and molecular adaptations such as collateral blood vessels and heat shock proteins, for example. Thus extra coronary arteries would increase lifespan despite age-related atherosclerosis, hypertension and thrombosis. Aging-tolerance is a concept that can solve some mysteries of aging. Many potential questions that might be asked are purely medical. Their answers can be found in medical textbooks.

There are a few questions that are difficult to answer now:

1. What TOR-independent pathways contribute to hyperfunctional aging? For example, sirtuins, FOXO, AMPK and IGF-1 can all be linked to the mTOR networks [201-206]. What about JNK [158, 207-209] and NF-kB [210], [162, 211, 212]? Are these pathways TOR-independent? And what are crucial downstream effectors of TOR that control aging?
2. It seems that rapamycin should be used in intermittent fashion, perhaps in combinations with e.g. metformin, lipid-lowering drugs, and beta-blockers and angiotens together with dietary restriction and physical exercise. But what are the exact doses and schedules maximize positive and minimize negative effects?
3. What would be the causes of death if TOR-driven aging were suppressed? Hyperfunction driven by run on of other pathways? Accumulation of molecular damage? Mitochondrial expansion? Other types of unknown aging? Would anti-oxidants become useful for that types of aging? And what are the pathological manifestations of accumulation of random molecular damage?

## The peculiar role of molecular damage

**Figure 7:**
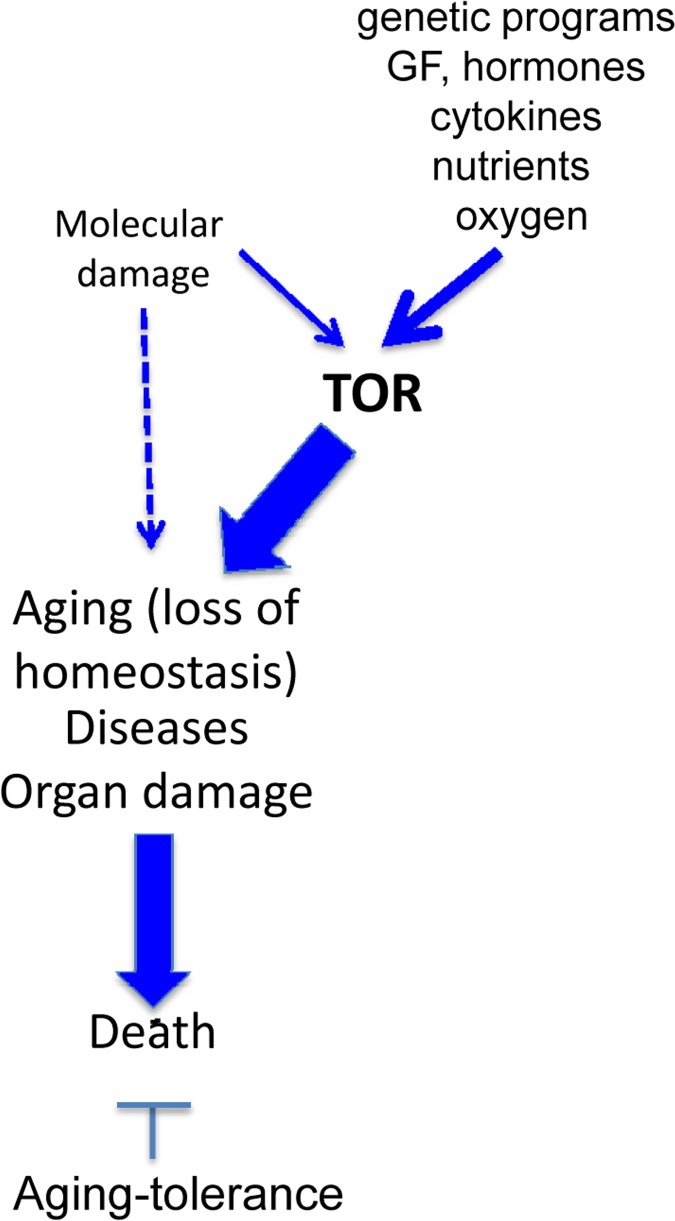
Incorporation of a hypothethetical role of molecular damage in the hyperfunction theory

Repair of random molecular damage is so important that cumulative damage does not reach a deadly threshold during the lifetime. In progeria [213], fitness is low from day one. There is a very strong natural selection for repair and maintenance. In contrast, mTOR-driven functions are essential early in life and there is a very strong selection for robust mTOR-dependent functions, even if their continuation (hyperfunctions) are harmful in old age. Still, we cannot exclude contribution of molecular damage to some symptoms of aging (Fig.7). This is simply unknown. May it decrease aging-tolerance [127, 214]? This is a fascinating question to answer.

A peculiar case is cancer. Accumulation of damage does not make a cancer cell fragile, arguing against the molecular damage theory, but instead via rounds of selection and proliferation which create robust cells that damage the organism. Notably, such selection of random mutations culminates in non-random activation of the mTOR pathway [122]. Activation of the PI3K/mTOR pathway is the most common alteration (and therapeutic target) in cancer [215-227]. Also, hyper-activation of the DNA damage response, involving TOR-like kinases, may contribute to hyperfunction. Therefore, molecular damage and autonomous activation of damage-sensing signal-transduction pathways may contribute to hyperfunction, not *vice versa*.

## The last argument for molecular damage theory

The last argument by Zimniak is: “Hyperfunction is one of several sources of molecular damage, on equal footing with reactive metabolites, toxicants, ROS, electrophiles, stochastic events, and many others” [3]. This argument will not be discussed all over again. Not only because hyper-functions are not a source of accumulation of molecular damage. But mostly because the starting point of this article is that the theory of molecular damage did not fit numerous observation, made incorrect predictions, did not contribute to medical advances, and did not lead to any practical application. As philosophers teach us, the theory cannot be wrong (or right), but it can be useless.
